# “Dendroarchitectonics”: From Santiago Ramón y Cajal to Enrique Ramón-Moliner or vice versa?

**DOI:** 10.1007/s10072-022-06151-3

**Published:** 2022-06-08

**Authors:** Felix Geser, Johannes Haybaeck, Deniz Yilmazer-Hanke

**Affiliations:** 1 Department of Geriatric Psychiatry, Klinikum Christophsbad, Faurndauer Str. 6-28, 73035 Göppingen, Germany; 2 Department of Pathology, Neuropathology and Molecular Pathology, Medical University of Innsbruck, Innsbruck, Austria; 3 Diagnostic & Research Center for Molecular Biomedicine, Institute of Pathology, Medical University of Graz, Graz, Austria; 4 Clinical Neuroanatomy, Department of Neurology, University Hospital, Ulm University, Ulm, Germany

**Keywords:** Dendroarchitectonics, Phylogeny, Ontogeny, Santiago Ramón y Cajal, Enrique Ramón-Moliner

## Abstract

Here, we review the morphological taxonomy of neurons proposed by Enrique Ramón-Moliner in the vertebrate central nervous system based on "dendroarchitectonics" and compare these findings with Santiago Ramón y Cajal's work. Ramón-Moliner distinguished three main groups of nerve cells situated on a spectrum of dendritic configuration in the mammalian central nervous system with decreasing degree of morphological specialization, i.e., idiodendritic, allodendritic, and isodendritic neurons. Leptodendritic neurons would be an even more primitive type, and lophodendritic nerve cells would develop into pyramidal neurons. Using two developmental lines (i.e., telencephalic and rhombencephalic trends), Ramón-Moliner reconstructed the probable course of events in the phylogenetic history that led to the dendroarchitectonic families. While an increasing morphological specialization is associated with the projected phylogenetic development as an abstract "whole," phylogenetically "primitive neurons" such as the reticular formation may be present in later phylogenetic stages, and vice versa, phylogenetical "new arrivals," such as the cortical pyramidal cell, may be found early in phylogeny. Thus, Ramón-Moliner adopted the notion of an in-parallel neuronal development during phylogeny and ontogeny. In contrast, Cajal argued earlier in favor of the idea that ontogeny recapitulates phylogeny, focusing on the pyramidal neuron. In ontogeny, the early developmental features show a higher degree of similarity than the comparison of their adult forms. These results corroborate the rejection of the interpretative framework of ontogeny as a simple, speedy repetition of the phylogeny. Understanding morphological findings with the change in their interpretation and the historic underpinnings provide a framework for refined scientific hypotheses.

## Introduction

**Fig. 1:**
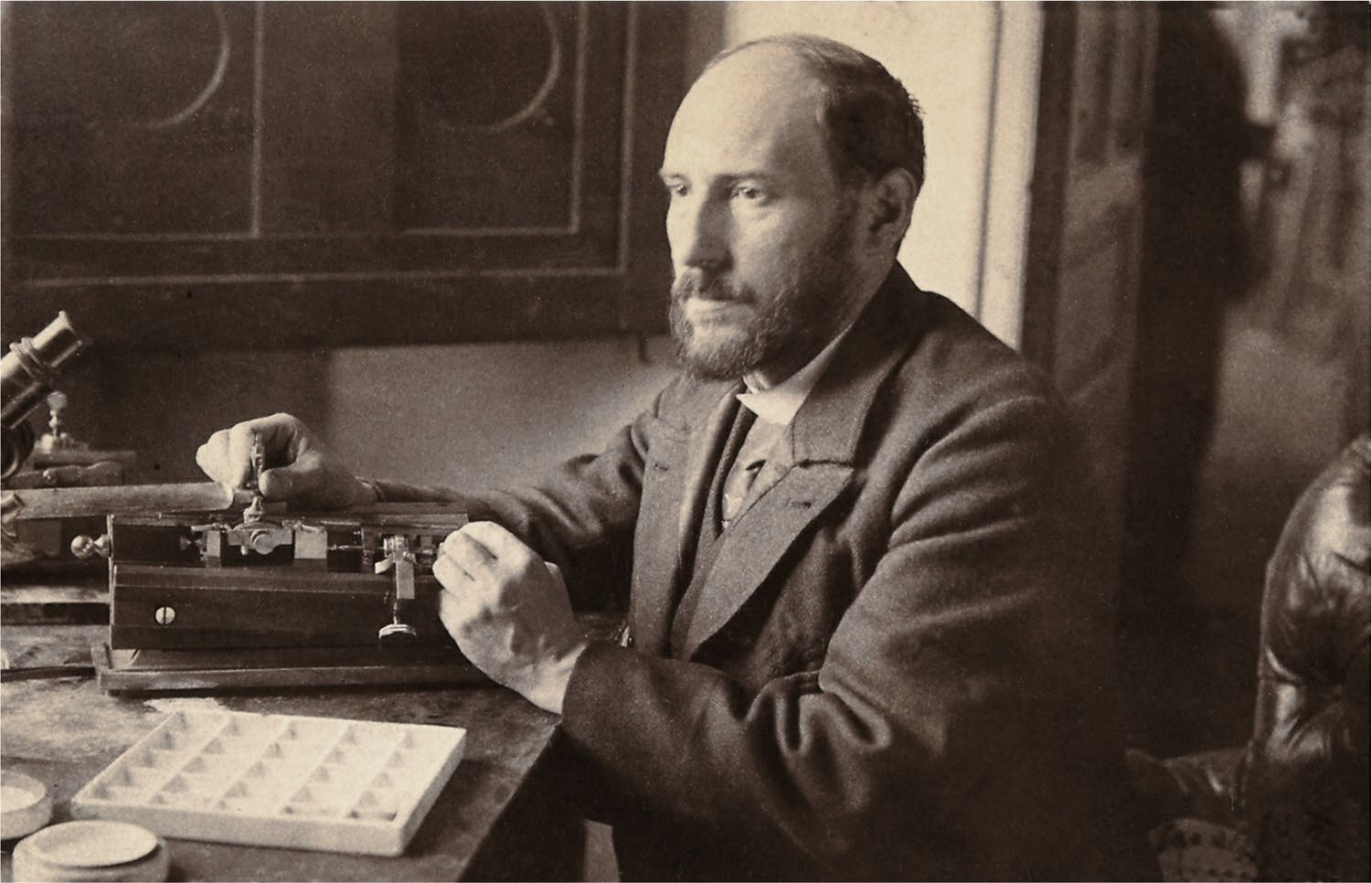
Santiago Ramón y Cajal (1852-1934). Courtesy of Cajal Legacy. Instituto Cajal (CSIC), Madrid

**Fig. 2:**
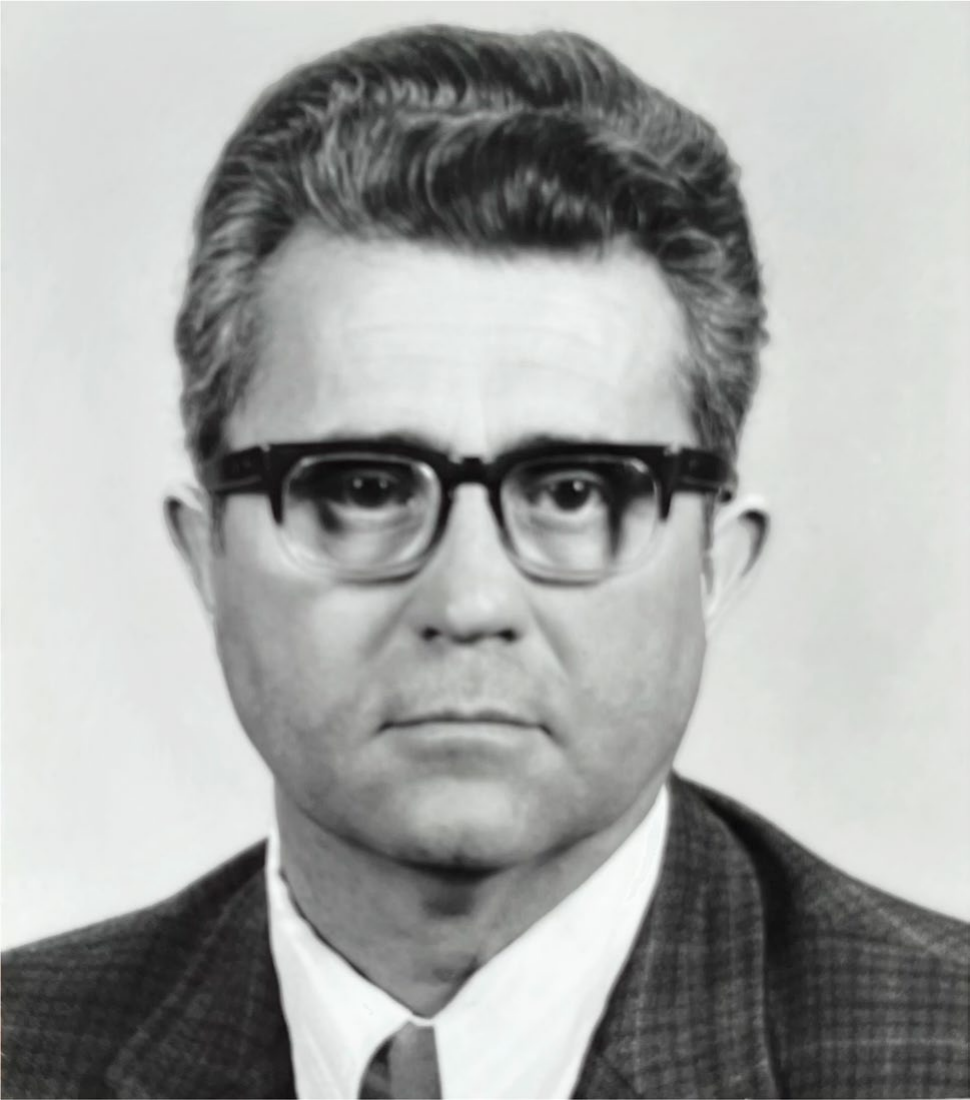
Photograph of Professor Enrique Ramón-Moliner (1927-1999), reproduced with permission from his family. Spanisch born E. Ramón-Moliner earned his Bachelor in Medicine in Madrid in 1951. From 1951 to 1956, he worked as a Research Fellow at the Ramón y Cajal Institute in Madrid, where he graduated as a Doctor of Medicine in 1956. From 1956 to 1959, he was employed as a Research Fellow at the Montreal Neurological Institute, McGill University (Québec, Canada), where he received his PhD degree. He held the position of Assistant Research Professor of Anatomy at the University of Maryland, Baltimore (USA), from 1959 to 1963. From 1963 to 1968, he served as an Associate of the Medical Research Council (MRC) of Canada and Assistant Professor at the Department of Physiology, Laval University (Québec, Canada). In 1968, the MRC associateship was transferred to Sherbrooke University (Québec, Canada). In the same year, he was appointed Associate Professor at the Department of Anatomy, Sherbrooke University, where he was promoted to Professor in 1974. In 1970, he also took extended travel leave as an MRC associate to continue his studies at the Department of Anatomy, Boston University, Cambridge, Massachusetts, USA, and at the Department of Anatomy, Aarhus University, Denmark

The fundamental neuroanatomical work of Santiago Ramón y Cajal (Fig. 1) from around the turn of the nineteenth to the twentieth century, such as his influence on the neuron theory and the idea of nervous system plasticity, is still highly relevant [1–4]. One of his favorite topics was the scientific question of what makes the human cerebral cortex with its pyramidal cells special and different from other species and how ontogenetic development relates to phylogeny. Thus, he and many others carried out comparative neuroanatomical studies to solve the old question of the structural basis of cognitive and mental skills such as creativity, which is still under debate [5–12]. Indeed, the multilayered neocortex allows for complex information processing with intricate abilities such as cognitive or motor functions [13]. More than half a century after S. R. y Cajal's work, Enrique Ramón-Moliner (Fig. 2), adopting the same morphological perspective and studying various species' nervous systems, arrived at a different model in terms of an integrative phylogenetic-ontogenetic interpretative framework. Understanding the findings that have led to this change serves as the basis for generating future scientific hypotheses. Thus, we discuss these morphological findings and their implications on ontogenetic development and phylogenetic history. For the presentation of the relevant work of S. R. y Cajal, we use the annotated and edited translation from Spanish—with the additions of the French version—into English by P. and T. Pasik. In keeping with this, S. R. y Cajal 1904 (vol. 2, second part) corresponds to Pasik and Pasik, 2002 (vol. 3).

## The cortical comparative anatomy and histogenesis of S. R. y Cajal

When describing the olfactory apparatus, S. R. y Cajal distinguished the first-order olfactory center, the olfactory bulb, where the central processes of the olfactory mucosa's bipolar collector cells terminate. The second- and third-order olfactory centers comprise the lateral olfactory tract, anterior olfactory nucleus/frontal cortex subjacent to the lateral olfactory tract, parahippocampal gyrus/pyriform lobe, including the (medial) entorhinal cortex, (pre-)subiculum, and others. With the fourth-order olfactory centers, S. R. y Cajal distinguished a hippocampal gyrus from a dentate gyrus (for their layers, see Table 1) [14]. Accordingly, the hippocampal gyrus is a thin and simplified band of the cerebral cortex. Its free border apparently is covered by the cavity of the dentate gyrus, which is an even more simplified cortical structure. These investigations were built upon earlier studies, such as the work of Sir Grafton Elliot Smith (1871–1937), who was a distinguished Australian anatomist interested in Egyptian mummies and paleoneurological diseases [15, 16]. Early in his professional life, in 1896, he demonstrated that in lower mammals (such as the platypus), there are regions of the hippocampal gyrus where the zone of granules of the dentate gyrus appears in continuation with the hippocampal pyramidal cells [17].

**Table 1:** Cortical layer structures

Dentate gyrus	Outer plexiform or molecular zone	
	Zone of granules (corresponding to the pyramidal cells of the cerebrum)	
	Zone of polymorphic cells	
Hippocampal gyrus	Epithelial or ependymal layer	
	Alveus or white matter	
	Stratum oriens or inner plexiform layer	
	Layer of the hippocampal pyramidal cells (corresponding to the large and small pyramidal cells of the prototype cortex)	
	Stratum radiatum or intermediate plexiform zone	
	Layer of horizontal fibers or stratum lacunosum	
	Molecular or outer plexiform layer	
Cerebral cortex	Amphibians (such as the frog, salamander, or triton)	Epithelial zone (ventricular ependyma)
		Zone of granules or pyramidal cells
		Molecular layer/plexiform zone
	Reptiles (such as the chameleon or lizard), dorsomedial area	Ventricular ependyma
		White matter
		Inner/deep plexiform layer
		Layer of pyramidal cells
		Outer/superficial plexiform layer
	Birds	Ependymal layer
		Layer of inner/deep stellate cells
		Layer of large pyramidal and stellate cells
		Layer of small/superficial stellate cells
		Molecular or plexiform layer
	Small mammals such as rodents (mouse, rat, rabbit, guinea pig)	Ependyma
		White matter
		Layer of ovoid or polymorphic cells
		Layer of large pyramidal cells
		Layer of medium pyramidal cells
		Layer of small pyramidal cells
		Molecular/Plexiform layer
	Humans and other gyrencephalic mammals (such as the monkey, dog, or cat)	Ependyma
		White matter
		Fusiform cell layer
		Inner medium pyramidal cell layer
		Inner large pyramidal cell layer
		Dwarf pyramidal cell and stellate cell layer (or granule cell layer)
		Outer medium and large pyramidal cell layer
		Small pyramidal cell layer
		Plexiform layer (layer poor in cells of Meynert/molecular layer of many authors)

Adapted from [14]

S. R. y Cajal and other authors at his time explored the comparative anatomy of the cerebral cortex (Table 1). He specifically commented in detail on fish, amphibians, reptiles, birds, and small mammals [14]. In fish, the presence of a pallium or cortex was, at least generally, denied. They only have the basal region of the cerebrum, corresponding to the pyriform lobe, parolfactory regions, and corpus striatum of higher vertebrates. The cerebral cortex of amphibians would be a most simple construction, representing a rudiment of the hippocampal gyrus. It consists of three fundamental layers: the zone of epithelial cells, the zone of granules or pyramidal cells, and the molecular or plexiform zone. The latter is the thickest and comprises two formations: first, the dendritic plexus, which is characteristic of the plexiform or molecular layer of humans and higher vertebrates and occasionally contains neurons (layer poor in cells of Meynert); second, the projecting and association axons. This superficial arrangement of fibers recalls that of the funiculi of the spinal cord. Although less accentuated, this array can also be seen in certain regions of the mammalian cerebrum, such as the extrinsic fibers of the hippocampal and dentate gyri or the lateral olfactory tract covering the temporal cortex. In reptiles, the axon of the pyramidal cell turns toward the inner surface of the brain and gives off collaterals below instead of above the soma. The cerebrum of birds shows an enormous size of the corpus striatum or fundamental ganglion, which is adherent to the cortex proper except in the medial aspect of the hemispheres. In this area, a prolongation of the ventricle separates these two central nervous system (CNS) structures. The cerebral cortex of birds is composed of the ependymal layer, the layer of inner stellate cells, the layer of large pyramidal and stellate cells, the layer of small stellate cells, and the molecular or plexiform layer. The cerebrum of small mammals such as rodents would include, in addition to the ependyma and white matter, the following cortical zones: the layer of ovoid or polymorphic cells, layer of large pyramidal cells, layer of medium pyramidal cells, layer of small pyramidal cells, and the plexiform layer. This layer array is a simplification of the cortex in humans and other gyrencephalic mammals (which are mammals with a folded cortex). These also show a granular zone, and their cerebrum includes (when leaving aside regional differences) the following layers in addition to the ependyma and white matter: fusiform cell layer, inner medium pyramidal cell layer, inner large pyramidal cell layer, dwarf pyramidal cell and stellate cell layer (or granule cell layer), outer medium and large pyramidal cell layer, small pyramidal cell layer, and the plexiform layer (layer poor in cells of Meynert, molecular layer). The granular cell layer shows massive growth in the human cortex compared to other gyrencephalic mammals [14].

According to S. R. y Cajal, the morphological simplification, beginning mainly in rodents and peaking in birds, reptiles, and amphibians, applies to the number of layers or the numbers of differentiated regions and, in particular, to the morphological features of nerve cells with dedifferentiation into this direction [14]. However, the neuronal radial orientation (with tufts emerging from the outward pole of the pyramidal cells) and the plexiform layer (with the tufts contacting with afferent fibers) would be a constant feature. With the extrapolated, high activity in the order of function of the pyramidal cell, he introduced the name "psychic cell," also called "psychomotor cell" [2], for these neurons. As to the cerebral cortex, any pyramidal neuron or other long-axon cell with a radial stem sends a dendritic tuft of ramification to the plexiform layer, but most short-axon neurons do not have a dendritic representation in the first layer [14]. S. R. y Cajal contended that the short-axon cells would function as "electric condensers of neural energy," transforming latent into active energy by an arriving current via an afferent fiber. This "dynamic reserve" would be the basis for outstanding human mental abilities and neural processes, which happen later than external excitation, such as memory or ideation [6, 14]. S. R. y Cajal considered the bitufted cells, a form of short-axon cells, as one of the essential features of the human cortex, given their high abundance; he found these cells rarely in dogs and cats, with less delicate features than humans.

**Fig. 3:**
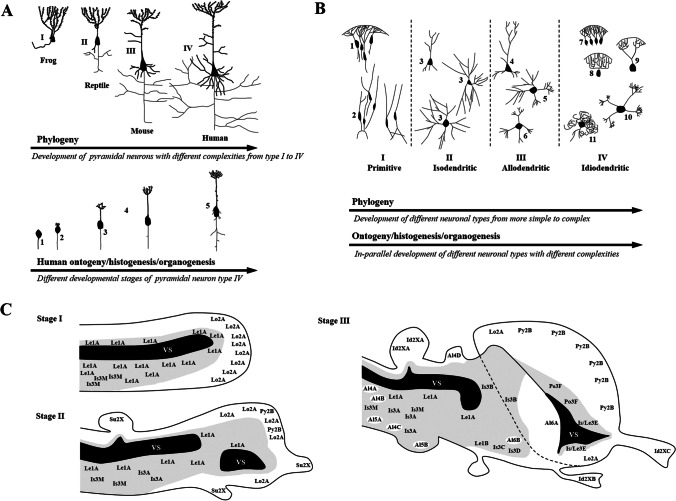
*A, upper tiers of the two schematic representations:* pyramidal cells in adult vertebrates (I, frog; II, mouse; III, reptile, IV, human). Mammalian ontogenetic development (1, neuroblast; 2, pyramidal cell in bipolar phase; 3, pyramidal cells with dendritic tufts; 4, pyramidal cell with a basal dendrite; 5, pyramidal cell with axon collaterals and branches of the apical dendrite). Redrawn with modifications from [14] with courtesy of publisher Springer Nature. *A, the respective lower tiers to the two schematic representations:* graphic illustration of S. R. y Cajal's idea that stages in the human ontogeny would correspond sequentially to the adult form of various vertebrates employing two arrows. *B, upper tier:* I. Neurons with subpial tufts/lophodendritic cells and periventricular leptodendritic neurons. II. Isodendritic neurons. III. Allodendritic neurons. IV. Idiodendritic neurons include subpial tufts/lophodendritic cells. All cells are schematic examples (1, neurons with subpial tufts; 2, leptodendritic neurons; 3, isodendritic pool; 4, pyramidal neurons with basilar dendrites; 5, allodendritic neurons of the diencephalon; 6, allodendritic neuron of the rhombencephalon; 7, tufted granule cells of the dentate gyrus; 8, Purkinje cells; 9, mitral olfactory neurons; 10, tufted neurons of various secondary sensory center; 11, wavy precerebellar neurons). The telencephalic trend may be responsible for the appearance of pyramidal neurons, the Purkinje cells, or olfactory mitral cells. The rhombencephalic trend is characterized by the transition from isodendritic to idiodendritic neurons. Redrawn with modifications from [20] with permission granted by Elsevier Science & Technology Journals. B, lower tier: graphic illustration of E. Ramón-Moliner's idea that ontogeny is not a mere recapitulation of the phylogeny employing two collateral arrows. *C, Stages I–III:* three-stage scheme of the reconstructed phylogenetic history of the central nervous system. Stage I means the primordial model, stage II denotes the progressive separation between the periventricular and subpial layers, and stage III is the final pageantry. Gray color indicates a pool of non-differentiated leptodendritic or isodendritic neurons (reticular formation), diffusely distributed in ill demarcated areas, in which more specialized neurons (allo- or idiodendritic) are embedded. The scheme "is based on the assumption that ontogeny does not necessarily recapitulate phylogeny" (see [18], p. 99). Le1A, leptodendritic neurons in periventricular regions; Le1B, leptodendritic hypothalamic groups; Lo2A, lophodendritic subpial neurons of the primitive cortex; Py2B, pyramidal neurons of the isocortex; Is3A, isodendritic regions of the rhombencephalon (reticular formation); Is3M, isodendritic (lower) motor neurons; Is3B, isodendritic regions of the diencephalon (perithalamic, intrathalamic, intralaminar, subthalamic); Is3C, isodendritic palestriatal groups; Is3D, prodiencephalic isodendritic regions (basal regions of the prosencephalon); Is/Le3E, septum (various leptodendritic septal nuclei, also the isodendritic medial septal nucleus); Po3F, deep polymorphic layers of the cerebral cortex; Al4A, allodendritic gracile and cuneate nuclei; Al4B, allodendritic sensory nucleus nervi trigemini; Al4C, allodendritic/idiodendritic cochlear nuclei; Al4D, allodendritic colliculi inferiors of the lamina quadrigemina; Al5A, allodendritic/idiodendritic inferior olivary nuclei; AL5B, allodendritic pontine nuclei; AL6A, allodendritic specific nuclei of the thalamus; AL6B, allodendritic specific nuclei of the neostriatum; Su2X, hypothetical neurons with subpial tufts, which could have developed into more specific types; Id2XA, idiodendritic Purkinje cells; Id2XB, idiodendritic retinal ganglion cells; Id2XC, idiodendritic olfactory bulb mitral cells. VS, ventricular system. Redrawn with modifications from [18, 21] with permission granted by Wolters Kluwer Permissions and courtesy of Pisa University Press (Archives Italiennes de Biologie).

Through investigations of pyramidal neurons in the cerebral cortex of animal series, embryonic and juvenile stages in the mammalian histogenesis, S. R. y Cajal postulated correspondence between the phylogeny and ontogeny [4, 14] (Fig. 3A). He generalized this idea to neuroglial cells. Based on structural similarities of "adult" forms of various vertebrates and mammalian ontogenetic stages, he contended that a given ontogenetic phase of the pyramidal neurons represents a phylogenetic phase. In keeping with this, the different forms of developing human neurons would be close to the "adult" forms of amphibians and reptiles. Figure 3A  shows the stages of the ontogenetic development of mammals and the adult forms of the pyramidal cells from various vertebrates (frog, reptile, mouse, and human). For the ontogenetic development of humans (Fig. 3A), he discriminated a neuroblast phase (neurons with an axon processing from the cell body), followed by the (secondary) bipolar phase (cells with a thicker, varicose process from one pole and a finer process emerging from the other pole). Pyramidal cells with basilar dendrites, axon collaterals, and centripetal fibers follow. However, some ontogenetic stages (e.g., bipolar cells) would not correspond to a phylogenetic phase since the individual development is characterized by a continuous development with more transitional structures. In phylogeny, some forms would be present rather transiently [14].

## The dendroarchitectonics of E. Ramón-Moliner

### Three principal neuronal types in the mammalian nervous system

According to the degree of morphological specialization, E. Ramón-Moliner proposed a taxonomy of nerve cells based on studies mainly of dendritic patterns, i.e., dendroarchitectonics [18–23]. He distinguished three main groups of nerve cells situated on a spectrum of dendritic configurations in the mammalian CNS. These include isodendritic, allodendritic, and idiodendritic neurons (Table 2, Fig. 3B). E. Ramón-Moliner based this classification on the dendritic structures' topognostic value. Isodendritic neurons show the lowest and idiodendritic the highest topognostic value, and allodendritic neuronal cells hold an intermediate position. Several subvarieties within these three dendroarchitectonic groups correspond to additional intermediary forms. Various neuronal regions often contain more than one type of neuron. A dendritic configuration with a low topognostic value denotes the difficulty of identifying an area in the CNS to which a given group of neurons belongs due to a lack of distinctive regional dendritic characteristics. The most commonly encountered isodendritic, generalized, or reticular neurons show a minimal variation of their features. These nerve cells typically show very long, relatively straight dendrites, not very rich in spines. Their dendrites either diverge or radiate from the cell body in all directions or show a slight tendency to orient themselves along given planes or dipoles. The primary dendritic segments (i.e., dendrites that arise directly from the cell body) are shorter than the secondary ones. Again, the secondary dendrites are shorter than the tertiary ones. Neuronal cells with this dendritic configuration are often associated with polygonal or triangular perikarya and a substantial degree of dendritic overlapping. In contrast, when the dendritic pattern is so distinct that the corresponding neuronal type can serve as a marker of a given region of the nervous system, there is a high topognostic value. The area to which these nerve cells belong can be directly identified. Indeed, there are neurons with more or less wavy or tufted dendrites. They exhibit very characteristic features and are referred to as allodendritic or idiodendritic, depending on their degree of differentiation or specialization, considering their dendritic morphology, connection, and functions. The appearance of idiodendritic neurons is so bizarre and striking that the observer can identify these regions at a glance based on a single cell. They display a high level of tuftedness or waviness, constituting an extreme pole opposite the isodendritic neuron. "Monopolization," typical for most idiodendritic nerve cells, means the relative exactness of their afferent or efferent contacts. Allo-/idiodendritic neurons appear very early in embryonic life, long before invasion by afferent fibers. E. Ramón-Moliner introduced the term "isodendritic core," which means a formation of isodendritic cells, not including "motor" or "sensory" cells by common definition [18, 23]. The isodendritic cells form a vast continuum of overlapping dendritic fields extending from the spinal cord, the rhombencephalon, mesencephalon, and diencephalon to the basal forebrain. These fields show diffuse afferent and sometimes efferent connections. Thus, the isodendritic core corresponds to—with certain limits—an area usually denoted by the reticular formation. As E. Ramón-Moliner stressed, any well-outlined center, i.e., "nuclei," must be excluded from the isodendritic formation, and only "regions" or "areas" with no actual boundaries should be subsumed under the isodendritic core. Thus, the aggregations of overlapping dendritic neurons form some "filling nervous system" or matrix encasing the specialized centers. For communication reasons, parcellating vocabularies, such as the one proposed by Olszewski and Baxter for the human brain stem and that of Taber for the brain stem of the cat [24, 25] were considered useful.

**Table 2:** Three principal neuronal types in the mammalian central nervous system

Isodendritic territories/isodendritic core	Spinal cord regions	Motoneuronal centers, nucleus proprius (pars medialis) of the spinal trigeminal nucleus
	Brainstem regions (medulla oblongata pons, mesencephalon)	Nucleus of the solitary tract, area centralis medullae oblongatae, area gigantocellularis medulla oblongatae, area paragigantocellularis dorsalis, area parvocellularis medulla oblongatae, area paramediana medulla oblongatae, nucleus prepositus hypoglossi, paramedian nuclei and intrafascicular nucleus of the hypoglossal nerve, lateral reticular nucleus, ventral cuneate nucleus, region of the nucleus raphe magnus, vestibular nuclei, area centralis tegmenti pontis, area paralemniscalis tegmenti, region of the locus ceruleus/nucleus subceruleus, nucleus motorius nervi trigemini, nucleus nervi abducentis, ventral tegmental area of Tsai, area centralis superior tegmenti, area cuneiformis tegmenti, area subcuneiformis tegmenti, region of the nucleus interstitialis tegmenti, nucleus tegmenti pedunculopontinus pars oralis/pars compacta, area peripeduncularis tegmenti, region of the nucleus sagulum, nucleus nervi oculomotorii, nucleus nervi trochlearis, nucleus nervi facialis, lower layers of the mammalian superior colliculus, substantia nigra pars compacta and substantia nigra lateralis
	Perithalamic areas	Reticular nucleus of the thalamus, zona incerta
	Intrathalamic regions	Intralaminary nuclei of the thalamus
	Lateral habenular nucleus	
	Subthalamic areas	Fields of Forel/zona incerta, globus pallidus
	Hypothalamic regions/lateral mammillary nucleus	
	Medial and lateral basal forebrain areas	Lateral preoptic area or the substantia innominata
	Medial septal nucleus	
	Deep cerebellar nuclei	
	Deep polymorphic layer of the cerebral cortex	
Borderline between allodendritic and isodendritic	Nerve cells located in the medial mammillary nucleus	
Allodendritic neurons	Precerebellar neurons	Inferior olivary nucleus, lateral cuneate nucleus, Clarke’s column, pontine nuclei, the so-called lateral reticular nucleus, nucleus subtrigeminalis, pontine nuclei, the papillioform nucleus of the pontine tegmentum (pterygoid nucleus), and the corpus pontocuneatus
	Prethalamic relay centers	Most dorsal portions of the nucleus gracilis et nucleus cuneatus, principal and spinal nuclei of the trigeminal nerve
		Most of the centers believed to be involved in the auditory pathway (dorsal and ventral cochlear nuclei, superior olivary nuclei, nucleus of the trapezoid body, nucleus of the lateral lemniscus, colliculi inferiores)
		Retinal ganglionic neurons
	Neothalamic allodendritic neurons (“precortical thalamic type”)	Nucleus dorsomedialis and nucleus ventralis posteriorlateralis, nucleus ventralis lateralis, nucleus anterior ventralis, and lateral and medial geniculate body
	Striatal allodendritic types	Neurons of the caudate and putamen
	Basal and central nuclei of the amygdaloid complex and the claustrum	
	Cortical types of neurons (pyramidal neurons with basilar dendrites and others)	
	“Limbic allodendritic types”	Medial habenular nucleus, the interpeduncular nucleus, and the tegmental nuclei of Gudden in the cat
Mixed population of allo- and idiodendritic nerve cells	Nuclei implicated in the auditory pathway, the inferior olivary nucleus, lateral reticular nucleus, the paramedian nuclei, intrafascicular nucleus of the hypoglossal nerve	
Idiodendritic cells	Cerebellar cortex	Purkinje cells, granule cells
	Wavy neurons in precerebellar cells	
	Retinal ganglionic cells	
	Mitral cells of the olfactory bulb	
	Tufted neurons in various secondary sensory centers	

Adapted from [18, 21, 23]

Isodendritic territories include the following: spinal cord areas, brainstem regions with the medulla oblongata, pons, and mesencephalon, perithalamic areas, intrathalamic regions, lateral habenular nucleus, subthalamic areas, hypothalamic regions/lateral mammillary nucleus, medial and lateral basal forebrain areas, medial septal nucleus, deep cerebellar nuclei, and the deep polymorphic layer of the cerebral cortex (Table 2). Considering the similarities between the histologic features of the isodendritic core and the relatively disorganized nervous system of lower vertebrates, E. Ramón-Moliner postulated that the isodendritic core would represent, from the perspective of phylogeny, a pool of pluripotential nerve cells. The lack of morphological specialization often manifests pluripotential. Throughout phylogeny, the cells of the isodendritic core remained relatively undifferentiated, diffusely distributed in the brainstem and other CNS areas. The degree of dendritic complexity and differentiation is generally a manifestation of phylogenetic development, probably due to being monopolized by specific functions and connections of certain cells. Isodendritic cells are often involved in processing afferent signals of a very heterogeneous source. The usually more demarcated allodendritic and idiodendritic cell groups would have become segregated from these, increasing the topognostic value. E. Ramón-Moliner stressed that the postulated sequence of events should be considered a phylogenetic, not ontogenetic, process. Most of the mammalian brainstem sensory relay neurons show a more or less specialized dendritic configuration. Specialized, allodendritic neurons are found in the specific precerebellar regions and prethalamic relay centers. There are also neothalamic allodendritic neurons ("precortical thalamic type"). Striatal allodendritic types include the caudate and putamen neurons. The central nuclus of the amygdaloid complex shows a dendritic pattern that apparently belongs to the same dendroarchitectonic family as the neostriatum. These regions originate from a basal telencephalic anlage which may account for the dendroarchitectonic similarity. E. Ramón-Moliner described "limbic allodendritic types," which appear to be relay stations as a sort of "centrifugal limbic lemniscus" situated adjacent to the reticular formation. The dendritic configurations of nerve cells in the medial mammillary nucleus are borderline allodendritic–isodendritic [20].

Cortical types of neurons (pyramidal neurons with basilar dendrites and others) are also included in the category of allodendritic neurons. As E. Ramón-Moliner stressed, the notion that the cerebral cortex is indispensable for the most elaborate patterns of behavior and function does not implicate that the pyramidal neuron, probably being the most frequently encountered neuronal type in man's CNS, is a "newcomer" in the phylogeny. In fact, the pyramidal neuron with its apical dendrite represents one of the earliest trends in neuronal configuration. The nerve cells with processes orientated toward the pia mater, i.e., neurons with subpial tufts, are characteristic of the cerebral cortex. However, in lower vertebrates, they are not only present in the telencephalon but also in the brainstem. The considerable development of the basal dendrites with an expansion of the dendritic tree seems to be characteristic of mammalian pyramidal cells. The non-pyramidal neuron in the cerebral cortex belongs to a very heterogeneous group, often called "stellate cells" or "star cells," accumulating in the third and fourth layers of the cortex. These are neurons with dendrites radiating in all directions but without a typical apical dendrite. The average cell type of the second layer is the small pyramidal cell ("granule cell"), which cannot generate an apical dendrite due to the proximity to the pia mater. Short-axon cells (Golgi type II cells) are present in all layers of the cerebral cortex and many other CNS regions. The highly differentiated or specialized idiodendritic cells include cells of the cerebellar cortex (Purkinje cells, granule cells), retinal ganglionic cells, and the olfactory bulb mitral cells. Tufted neurons in various secondary sensory centers and wavy neurons in precerebellar nuclei are also idiodendritic neurons. The nuclei implicated in the auditory pathway, the inferior olivary nucleus, the lateral reticular nucleus, and the hypoglossal nerve's paramedian nuclei and intrafascicular nucleus are characterized by mixed populations of allo- and idiodendritic cells [20] (Table 2).

### Two main types of nerve cells in lower vertebrates

E. Ramón-Moliner distinguished two main types of nerve cells in the nervous system of lower vertebrates—see Table 3 and Fig. 3B (e.g., Lophius piscatorius in [20]). First, as already mentioned, neurons with subpial tufts (lophodendritic) and, second, leptodendritic neurons. These types are situated on opposite sides of a spectrum of intermediate forms, whereas in mammals, two extreme poles remain with the leptodendritic neurons located in periventricular positions. The dendritic pattern of lophodendritic neurons is reminiscent of neurons in the dentate gyrus of the mammalian hippocampus. The dendroarchitectonic configuration of the latter neurons with an absence of basilar dendrites is probably one of the most primitive patterns in the nervous system. The lophodendritic neurons have become pyramidal cells in mammals and, thus, represent a primitive type that changes very little in the course of phylogeny. These cells keep their bushy appearance and subpial location in a few circumscribed areas of the telencephalon. They would be the primitive prototype of neurons that, according to S. R. y Cajal, have developed toward the pyramidal neurons [14]. The dendritic configuration of leptodendritic neurons is reminiscent of those in the mammalian hypothalamus. In fact, E. Ramón-Moliner proposed the presence of a "leptodendritic core" lying within this isodendritic core of mammals, with primitive cells populating periventricular or subependymal regions of the diencephalon, mesencephalon, and rhombencephalon [19]. The leptodendritic neurons show only a few, although relatively long, poorly ramified dendrites and a conical or fusiform cell body. This type of neuron may represent a "living fossil", the prototype from which most of the nerve cells of the vertebrate CNS may have derived. Table 3 depicts the areas of the leptodendritic core. The hypothalamus probably shows the most striking examples of leptodendritic neurons in the mammalian CNS. E. Ramón-Moliner contended that the leptodendritic core of the diencephalon and rhombencephalon is the final point of convergence for the projections from the limbic system [20]. Since these territories show very little change in dendroarchitectonic configurations during phylogeny, the leptodendritic core could have an even more primitive significance than that of the surrounding isodendritic core. Indeed, one of the main differences between the tegmentum of higher and lower vertebrates is the proportion of leptodendritic and isodendritic neurons, with the latter being much more common in mammals. In fact, in the CNS of lower vertebrates, the leptodendritic neurons are the most widespread neuronal type. In line with the contention that the periventricular regions have an undifferentiated character, hypothalamic neurosecretory nuclei, the medial habenular nucleus, the subcommisural organ, and the area postrema are subependymal derivatives with both glandular and neural properties. This finding reflects the common neuroectodermal origin of glandular and neural structures.

**Table 3:** Two main types of nerve cells in lower vertebrates

Leptodendritic neurons	Neurons with only a few, although relatively long, poorly ramified dendrites and a conical or fusiform cell body	Slender cells populating periventricular or subependymal regions of the rhombencephalon, mesencephalon, diencephalon and others: area centralis medulla oblongatae, area parvicellularis medullae oblongatae, dorsal motor nucleus of the vagus, the nucleus of the solitary tract, area gigantocellularis, area paralemniscalis, nucleus tegmenti pedunculopontinus (pars oralis), pontomesencephalic central gray matter, nucleus vestibularis medialis, nucleus of Darkschewitch, nucleus of Edinger-Westphal, substantia nigra pars reticulata and pars compacta, hypothalamus, subthalamus, nucleus pallidus, various septal nuclei
Lophodendritic neurons	Neurons with an apical dendrite and subpial tufts (bushy appearance), (relative) absence of basilar dendrites	A few localized areas of the telencephalon including the granule cells of dentate gyrus, the taenia tecta, hippocampus/archipallum, and maybe other portion of the basal forebrain (area praepyriformis), some non-telencephalic regions of lower vertebrates

Adapted from [18, 19, 21] and [14]

### Reconstructed phylogenetic history of the nervous system

Based on the above mentioned, two main trends in the development of dendritic patterns were postulated [18, 21] (Table 4, Fig. 3B). First, as illustrated in S. R. y Cajal's work, a telencephalic trend refers to the development of those nerve cells with subpial dendritic tufts [14]. The telencephalic trend may be responsible for the appearance of pyramidal neurons of the cerebral cortex and may also be reflected in the subpial distribution of the Purkinje cells [18]. The position of dendritic tufts of neurons in sensory regions that originated as evaginations of the primordial prosencephalic vesicle, i.e., the retinal ganglionic and olfactory mitral cells, may reflect an extension of the telencephalic trend. Second, a rhombencephalic trend denotes a phylogenetic process evident preponderantly in, but not confined to, the rhombencephalon. This process is characterized by certain nerve cell groups segregating out of a primordial and pluripotential core due to a decrease in the general size of their dendritic fields and an increase in dendritic branching. This process means a transition from isodendritic to idiodendritic neurons. In keeping with these ideas, E. Ramón-Moliner reconstructed the probable course of events in the phylogenetic history that led to the dendroarchitectonic families observable in the whole mammalian brain [18, 20, 21] (Table 4, Fig. 3B, and Fig. 3C). The three-stage scheme assumes that "ontogeny does not necessarily recapitulate phylogeny" (see [18], p. 99). In the first stage (I), the primordial model, both types of neurons, i.e., the periventricular leptodendritic cells (reminiscent of those found in the periventricular areas of the mammalian brainstem) and subpial lophodendritic neurons of the primitive cortex (reminiscent of neurons in dentate gyrus neurons of the mammalian hippocampus), would be intermingled. In addition, motor neurons would be present (in the sense of lower motor neurons). In the second stage (II), with the progressive phylogenetic separation between the periventricular and subpial layers, the two dendroarchitectonic families would become displaced now occupying the hypothalamus and the cerebral cortex, respectively. The following cells would appear in addition to those present in stage I: pyramidal neurons with well-developed basal dendrites in the neopallium ("isocortex"), Purkinje cells of the cerebellum, retinal ganglionc cells, olfactory bulb mitral cells, and isodendritic regions of the rhombencephalon. As a third stage (III) with the final dendroarchitectonic pageantry as the result of the two main trends in the probable development of dendritic configurations, the following cell groups would appear in addition: subthalamic nucleus (corpus Luysi), hypothalamus, regions of the diencephalon, paleostriatum, prodiencephalic areas (basal forebrain areas), septum, the deep polymorphic layer of the cortex cerebri, prethalamic neurons, precerebellar nuclei, as well as specific neurons of the thalamus, the neostriatum and the basal nuclei of the amygdala.

**Table 4:** Reconstructed phylogenetic history of the central nervous system

First stage (I), the primordial model	Leptodendritic neurons in periventricular regions	
	Lophodedendritic subpial neurons of the primitive cortex	
	Isodendritic (lower) motor neurons	
Second stage (II), progressive separation between the periventricular and subpial layers	Leptodendritic neurons in periventricular regions	Rhombencephalic trend: transition from leptodendritic/isodendritic to idiodendritic neurons predominantly in the lower brainstem, preservation of primordial/pluripotential core Telencephalic trend: development of neurons with subpial dendritic tufts (cortical pyramidal cells, Purkinje cells)
	Lophodedendritic subpial neurons of the primitive cortex	
	Isocortex (pyramidal cells with well-developed basilar dendrites of neopallium) Hypothetical neurons with subpial tufts, which could have developed into more specific types	
	Isodendritic motor neurons Isodendritic regions of the rhombencephalon (reticular formation)	
Third stage (III), the final dendroarchitectonic pageantry	Leptodendritic neurons in periventricular regions Hypothalamic groups	
	Lophodedendritic subpial neurons of the primitive cortex Isocortex Hypothetical neurons with subpial tufts, which could have developed into more specific types Idiodendritic Purkinje cells Idiodendritic retinal ganglion cells Idiodendritic olfactory bulb mitral cells	
	Isodendritic motor neurons Isodendritic regions of the rhombencephalon (reticular formation) Isodendritic regions of the diencephalon (perithalamic, intrathalamic, intralaminar, subthalamic) Isodendritic palestriatal groups Prodiencephalic isodendritic regions (basal regions of the prosencephalon) Isodendritic/leptodendritic neurons of the septum Deep polymorphic layers of the cerebral cortex	
	Allodendritic gracile and cuneate nuclei Allodendritic sensory nucleus nervi trigemini Allodendritic cochlear nuclei/idiodendritic ventral cochlear nucleus cells Allodendritic colliculi inferiors of the lamina quadrigemina	
	Idiodendritic/allodendritic inferior olivary nuclei Allodendritic pontine nuclei	
	Allodendritic neurons of the specific nuclei of the thalamus Allodendritic neurons of the specific nuclei of the neostriatum Allodendritic neurons of the specific nuclei of the basal amygdala	

Adapted from [18, 21] and [14]

## Discussion

Substantial advancement has been achieved in our scientific understanding of phylogeny and ontogeny over the past centuries, and the ideas about concerted vs. mosaic phylogenetic development (i.e., coordinated vs. independent variation) are still a matter of debate [26, 27]. In the 1960s and 1970s, E. Ramón-Moliner appreciated the impact of nervous function on the morphological specialization of cells leading to segregation and loss of diffuse (receptive) contacts [18, 20, 23]. S. O. E. Ebesson's "parcellation theory," raised in the 1980s, suggested that the nervous system becomes more complex during phylogeny, and likewise ontogeny, not by one system invading another but by parcellation (segregation–isolation) followed by selective loss of connections of the newly formed subsystems [28, 29]. Accordingly, overlapping circuits are more common in primitive (generalized) as compared to specialized brain organizations. He also stated that neocortical equivalents have been present since the start of vertebrate phylogenesis. However, brains may have less dense connections per se due to their increasing size [30]. Moreover, E. Ramón-Moliner stressed that, during phylogeny, a trend reversing the parcellation theory occurs [31]. For example, the (lower layer of the) colliculi superiores of mammals possess many heterogeneous connections with the reticular formation [23]; in contrast, in reptiles or amphibians, the superior colliculi have more demarcated borders, being more segregated. Herewith, he rejected the idea that the ontogenetic loss of connections always corresponds to adult ancestral forms.

Since humans and mice have homologous cells allowing for direct comparisons, studies on these animals have been used to investigate cortical development (e.g., [32]); however, these results cannot simply be transferred to humans [33]. The cortex of rodents, such as mice, has its own phylogenetic history. At the turn of the nineteenth to the twentieth century, S. R. y Cajal postulated a correspondence between phylogeny and ontogeny of the cerebral cortex, arguing that a given ontogenetic phase of the pyramidal neurons represents a phylogenetic phase [14]. He contended that ontogenetic phases of "the" cerebral pyramidal cell (the "psychic cell") would correspond roughly to "adult" forms as they phylogenetically appear in invertebrates/lower vertebrates. In contrast, more than half a century later, in reconstructing the probable course of events in the phylogenetic history that led to the dendroarchitectonic families employing a three-stage scheme, E. Ramón-Moliner did not consider ontogeny as a mere recapitulation of the phylogeny [18, 21]. Indeed, the lepto- or isodendritic configurations, the pools of undifferentiated neurons, remain diffusively distributed in the mammalian nervous system (such as the brainstem and periventricular areas of the cerebrum). Furthermore, the lophodendritic neurons and their development into pyramidal cells, characteristic of the cerebral cortex and indispensable for the most elaborate patterns of behavior and function, are no "new arrivals" but represent one of the earliest neuronal configuration trends. Neurons with subpial tufts are present not only in the telencephalon but also in the brainstem of lower vertebrates [20]. Patterning the neuraxis of vertebrates means a constant process over a long time in ontogenesis [34]. In terms of the terminal neurogenesis (i.e., the "birth" of cells following the final precursor cell division, yielding a neuroblast for further differentiation), various neurons with low morphological specialization, e.g., brainstem areas (raphe complex, superior colliculi, or locus ceruleus), and a high degree of morphological specialization (e.g., Purkinje cells) emerge at around the same in both monkeys and rats [35]; various limbic structures including hippocampus and amygdala show early development in monkey and synchronous neurogenesis with rats later on; and isocortical neurons terminally developed either at around the same time (layer VI), sequentially (layer V) or delayed (layer II-III) in monkeys as compared to rats. The earliest generated cell layers in the cerebral cortical development comprise preplate cells, with the Reelin secreting Cajal–Retzius cells being its most prominent component [36]. The latter cells are, in fact, a general feature of all vertebrates; however, there is an increasing Reelin signaling with developing cortical complexity, which might have contributed to the basic mammalian cortical pattern. The pallium in nonmammalian amniotes has a different architecture than its mammalian homolog, i.e., the six-layered neocortex; therefore, the capability to generate an orderly sequence of distinct neocortical cells was thought to have emerged in mammals [37]. However, it was shown that layer-specific neuron subtypes do exist in the chick pallium. Thus, the emergence of layer-specific neuron subtypes predates the development of the laminar architecture, suggesting that mammals and avians share the neocortical neuron subtypes [38]; also, their "common ancestor" may show a similar neuronal repertoire. Indeed, there is an astonishing degree of similarity, but not identicalness, in the features at the very early phases of development [39–41], whereas at later stages, brain development diverges in the various vertebrate groups [13, 27, 42–46]. The hypothesis of ontogeny as a "replay" of the phylogenetic development, i.e., phylogeny generating ontogeny with a highly conserved embryonic stage common to all vertebrates, is historically closely linked with the name Ernst Heinrich Philipp August Haeckel (1834–1919) [27, 30, 47, 48]; this hypothesis has also been referred to as the so-called biogenetic law. However, there are highly variable morphological features of vertebrate embryos forerunning essential differences in the adult structures [45]. More recent evidence also rejected the ontogeny recapitulating phylogeny idea by examining fishtails fossils [49]; instead, the final structure is ruled by differential growing.

## Conclusion

In ontogeny, early developmental features of different neuronal cell types show a higher degree of similarity than their adult forms' features. While an increasing morphological specialization is associated with the reconstructed, projected phylogenetic development as an abstract whole, phylogenetically "primitive neurons" such as those found in the reticular formation are present at later phylogenetic stages; conversely, phylogenetically "new neurons," such as the cortical pyramidal cell, may be found early during phylogeny. Thus, in higher mammals' adult nervous system, phylogenetically "old primitive" neurons can coexist with phylogenetically "new complex" neurons. These results corroborate the rejection of the interpretative framework of ontogeny as a simple, speedy repetition of the phylogeny. The re-interpretation of ontogenetic and phylogenetic similarities and differences of neuronal morphologies and the historic underpinnings thereof may provide a useful framework for refining scientific hypotheses.
