# Correction to: “Dendroarchitectonics”: From Santiago Ramón y Cajal to Enrique Ramón‑Moliner or vice versa?

**DOI:** 10.1007/s10072-022-06218-1

**Published:** 2022-06-21

**Authors:** Felix Geser, Johannes Haybaeck, Deniz Yilmazer‑Hanke

**Affiliations:** 1 Department of Geriatric Psychiatry, Klinikum Christophsbad, Faurndauer Str. 6‑28, 73035 Göppingen, Germany; 2 Department of Pathology, Neuropathology and Molecular Pathology, Medical University of Innsbruck, Innsbruck, Austria; 3 Diagnostic & Research Center for Molecular Biomedicine, Institute of Pathology, Medical University of Graz, Graz, Austria; 4 Clinical Neuroanatomy, Department of Neurology, University Hospital, Ulm University, Ulm, Germany

**Correction to: Neurological Sciences (2022)**

10.1007/s10072-022-06151-3

The above article was published online with missing gray zones in Fig. 3. The figure is now updated.

The Original article has been corrected.

